# Editorial

**DOI:** 10.1120/jacmp.v2i2.2613

**Published:** 2001-03-01

The Journal welcomes letters that have comments or questions about published articles. In most cases the author(s) of the article in question will be given the opportunity to reply. Both the letter and the reply will be published in the same issue.

I am very pleased to announce that Jim Smathers has joined the Editorial Board as the Book Review Editor. Book publishers and authors on medical physics and related subjects are encouraged to send copies of new works for review.

In addition, several other new features will be added to the Journal in the future. In particular we would like to encourage submission of short “Technical Notes” which should address a novel solution to a specific problem encountered in the practice of clinical medical physics. Construction or modification of equipment, special measurement techniques or theoretical analysis might be involved. Technical notes should only be a few pages long.

As this issue was being completed we learned of the death of John Wright. For many years John was an active member of the medical physics community and he served our profession well in a number of leadership roles. Among other honors he was a Fellow of the American College of Medical Physics. We shall miss his warm smile and enthusiastic spirit. To his wife, family, and many friends we send our heartfelt condolences.

Peter R. Almond, Ph.D.

Editor‐in‐Chief

